# Chronic respiratory disease disparity between American Indian/Alaska Native and white populations, 2011–2018

**DOI:** 10.1186/s12889-021-11528-8

**Published:** 2021-07-28

**Authors:** Kimberly G. Laffey, Alfreda D. Nelson, Matthew J. Laffey, Quynh Nguyen, Lincoln R. Sheets, Adam G. Schrum

**Affiliations:** 1 Department of Molecular Microbiology and Immunology, School of Medicine, University of Missouri, Columbia, MO USA; 2 Institute for Data Science and Informatics, University of Missouri, Columbia, MO USA; 3 Department of Surgery, School of Medicine, University of Missouri, Columbia, MO USA; 4 Department of Pathology and Anatomical Sciences, School of Medicine, University of Missouri, Columbia, MO USA; 5 Department of Health Management and Informatics, School of Medicine, University of Missouri, Columbia, MO USA; 6 Department of Biomedical, Biological, and Chemical Engineering, College of Engineering, University of Missouri, Columbia, MO USA

**Keywords:** BRFSS, American Indian/Alaskan Native, Chronic respiratory disease, Health disparities

## Abstract

**Background:**

American Indian/Alaska Native (AI/AN) populations have been disproportionately affected by chronic respiratory diseases for reasons incompletely understood. Past research into disease disparity using population-based surveys mostly focused on state-specific factors. The present study investigates the independent contributions of AI/AN racial status and other socioeconomic/demographic variables to chronic respiratory disease disparity in an 11-state region with historically high AI/AN representation. Using data from the Behavioral Risk Factor Surveillance System (BRFSS) spanning years 2011–2018, this work provides an updated assessment of disease disparity and potential determinants of respiratory health in AI/AN populations.

**Methods:**

This cross-sectional study used data from the BRFSS survey, 2011–2018. The study population included AI/AN and non-Hispanic white individuals resident in 11 states with increased proportion of AI/AN individuals. The yearly number of respondents averaged 75,029 (62878–87,350) which included approximately 5% AI/AN respondents (4.5–6.3%). We compared the yearly adjusted prevalence for chronic respiratory disease, where disease status was defined by self-reported history of having asthma and/or chronic obstructive pulmonary disease (COPD). Multivariable logistic regression was performed to determine if being AI/AN was independently associated with chronic respiratory disease. Covariates included demographic (age, sex), socioeconomic (marital status, education level, annual household income), and behavioral (smoking, weight morbidity) variables.

**Results:**

The AI/AN population consistently displayed higher adjusted prevalence of chronic respiratory disease compared to the non-Hispanic white population. However, the AI/AN race/ethnicity characteristic was not independently associated with chronic respiratory disease (OR, 0.93; 95% CI, 0.79–1.10 in 2017). In contrast, indicators of low socioeconomic status such as annual household income of <$10,000 (OR, 2.02; 95% CI, 1.64–2.49 in 2017) and having less than high school education (OR, 1.37; 95% CI, 1.16–1.63 in 2017) were positively associated with disease. These trends persisted for all years analyzed.

**Conclusions:**

This study highlighted that AI/AN socioeconomic burdens are key determinants of chronic respiratory disease, in addition to well-established risk factors such as smoking and weight morbidity. Disease disparity experienced by the AI/AN population is therefore likely a symptom of disproportionate socioeconomic challenges they face. Further promotion of public health and social service efforts may be able to improve AI/AN health and decrease this disease disparity.

**Supplementary Information:**

The online version contains supplementary material available at 10.1186/s12889-021-11528-8.

## Background

Asthma and chronic obstructive pulmonary disease (COPD) are prominent chronic respiratory conditions that incur significant health and financial costs in the United States [1, 2]. Both diseases entail airflow obstruction and airway inflammation that can progressively worsen and require long-term clinical management [3, 4]. Past surveillance and research efforts have shown the American Indian/Alaska Native (AI/AN) population to suffer a greater prevalence of these conditions [5–7]. The health disparities experienced by AI/AN peoples in various chronic diseases including respiratory diseases was shown to be associated with indicators of low socioeconomic status such as poverty [8, 9] and health risk behaviors such as tobacco use [10, 11]. These disease correlates are typically included and hence tracked in population-based surveys such as the Behavioral Risk Factor Surveillance System (BRFSS).

Yet additional correlates of chronic respiratory disease exist which may contribute to disease disparity observed in the AI/AN population. In particular, AI/AN children sustain higher rates of respiratory syncytial virus (RSV) infection [12] which is associated with development and/or exacerbation of asthma [13]. Further, persistent exposure to poor indoor air quality is also increasingly recognized to be associated with adverse respiratory health [14–16]. Lastly, AI/AN individuals were more likely to be exposed to occupational inhalants which adversely impact respiratory health [17–19]. These variables relate to the etiologies of asthma and COPD and are not included in the BRFSS. Consequently their potential contribution to explain the disease disparity is not known. In this study, we address the contributing influence of race/ethnicity (being AI/AN or non-Hispanic white) on disease status using the most recent multi-year BRFSS data. We hypothesize that if differences in disease etiology are associated with disease prevalence, their effect could be observed as a significant association between the race/ethnicity variable and chronic respiratory disease. Alternatively, if socioeconomic factors are the major drivers of disparity, then AI/AN race/ethnicity will not be independently associated with chronic respiratory disease after adjusting for socioeconomic covariates.

## Methods

We used data from the BRFSS survey conducted in the years 2011–2018. The BRFSS is an annual random-digit dialed telephone health survey of adult US residents aged 18 and older [20]. It is conducted across all US states and territories by state health departments in collaboration with the Center for Disease Control and Prevention. In 2017, the AI/AN population was oversampled in 11 states with historically high AI/AN relative populations to increase understanding of their health status [21]. These states were: AK, AZ, MN, MT, NE, NM, NC, ND, OK, SD, and WI. We therefore restricted our analysis to responses from residents of these 11 states. We generated a map showing the locations of these states and their overlap with federally recognized and statistical AI/AN entities by using the open source ‘tigris’ package [22] in R version 3.6.1. The shapefile containing the geographic information of these federally recognized AI/AN entities used for map plotting was obtained from the US Census Bureau [23]. The analysis of publicly available, de-identified data does not constitute human subjects research as defined in federal regulations, and thus this study did not require Institutional Review Board (IRB) review.

The primary outcome, chronic respiratory disease status, was dichotomized as Negative if respondents gave 0 affirmative answers for the following questions or as Positive if they gave at least 1 affirmative answer: ‘Have you ever been told you had asthma’ and ‘Have you ever been told you have chronic obstructive pulmonary disease, COPD, emphysema, or chronic bronchitis?’ The exposure variable, race, included AI/AN or non-Hispanic White (hereafter termed White) as indicated by the BRFSS computed race variable. Respondents of other races were not included in the analysis. The number of responses analyzed per year averaged 75,029 (62878–87,350) which included approximately 5% AI/AN respondents (4.5–6.3%).

Demographic, socioeconomic, and behavioral variables were included as covariates in the analysis. Demographic covariates with their respective levels included: sex (male, female); age (18–24, 25–34, 35–44, 45–54, 55–64, 65 and older). Socioeconomic covariates included marital status (currently married, never married, formerly married); education level (some high school and lower, high school graduate, some college, college graduate); annual household income (<$10,000, $10,000 - < $15,000, $15,000 - < $20,000, $20,000 - < $25,000, $25,000 - < $35,000, $35,000 - < $50,000, $50,000 - < $75,000, ≥$75,000, unreported income); and access-to-care (adequate, inadequate). Behavioral covariates included smoking status (smoker, non-smoker); and weight morbidity as defined by body mass index (BMI) (underweight, normal weight, overweight, obese). Access-to-care was a composite variable aimed to assess both healthcare access and utilization. It was dichotomized as Inadequate if respondents gave 0–1 favorable answers for the following questions or as Adequate if they gave ≥2 favorable answers: ‘Do you have any kind of health care coverage, including health insurance, prepaid plans such as HMOs, or government plans such as Medicare, or Indian Health Service?’, ‘Do you have one person you think of as your personal doctor or health care provider?’, and ‘Was there a time in the past 12 months when you needed to see a doctor but could not because of cost?’ Respondents were categorized as smokers if they gave a positive answer to either of the questions: “Have you smoked at least 100 cigarettes in your entire life?”, and “Do you now smoke cigarettes every day, some days, or not at all?”

Responses with missing values for any variables except annual household income were excluded from analysis. Due to a substantial proportion of responses with missing annual household income, this variable could not be assumed as missing at random. We therefore assigned these responses to an “unreported income” category and included it as a level during regression.

Bivariable and multivariable logistic regression analyses were performed on individual years of BRFSS data from 2011 to 2018. Rao-Scott χ^2^ test was used to test for statistical difference between categorical variables. 2-tailed *p* values < .05 are considered statistically significant. To select for significant covariates for the logistic regression models, a stepwise forward modeling process was used. Odds ratios indicate associations when confidence intervals (CI) exclude 1.

To account for the complex sampling design of the BRFSS, data analysis was performed with the survey package [24] using R version 3.6.1. Specifically, the yearly adjusted prevalence of chronic respiratory disease with 95% CI for AI/AN or white populations was calculated using yearly survey weights available as part of the BRFSS data.

## Results

### Prevalence of chronic respiratory disease

**Fig. 1:**
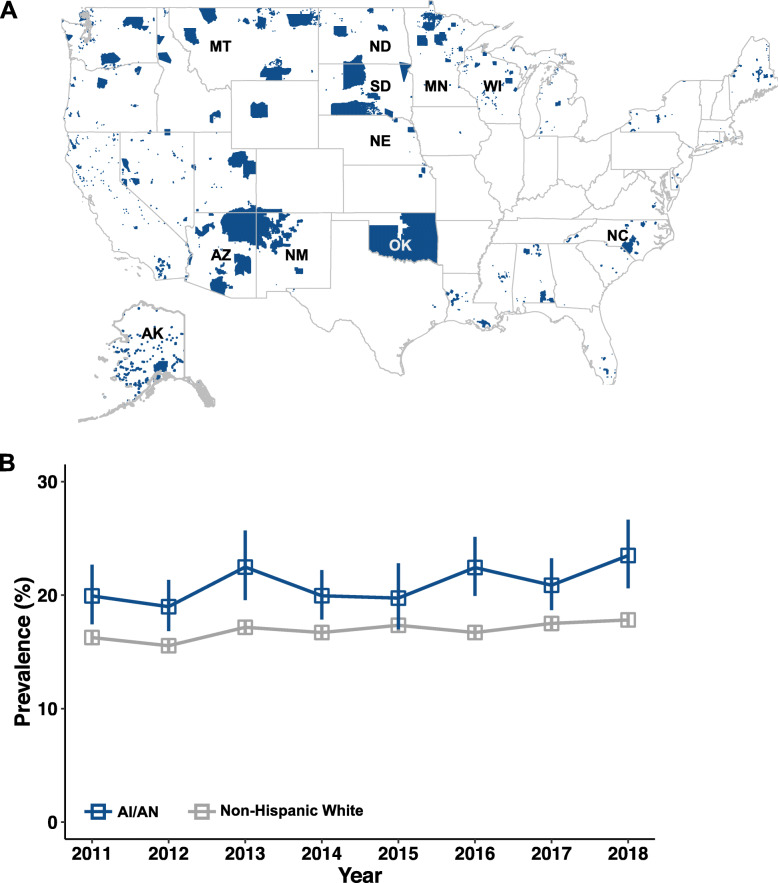
The AI/AN population experiences higher prevalence of chronic respiratory disease. **A** Location of federally recognized and statistical American Indian and Alaska Native entities in blue, including reservations. States included in this study are labeled with two-letter abbreviation and were oversampled in 2017 due to historically increased AI/AN representation. The map shown was produced using the tigris package [22] in R with shapefiles publicly available from the US Census Bureau [23]. **B** Adjusted prevalence of chronic respiratory disease in AI/AN and non-Hispanic white respondents surveyed in years 2011–2018. Comparisons between AI/AN vs white are statistically significant by χ^2^ test for all years except 2015

In 2017, oversampling for AI/AN respondents was done in 11 states identified by the US government as having historically higher AI/AN relative populations (Fig. 1A). Indeed, consistently for each year included in this study (2011–2018), about half of the US AI/AN population was resident in this 11-state group [25, 26]. To assess potential disparity in chronic respiratory disease status between AI/AN and white populations, we calculated the yearly weighted disease prevalence for 2011–2018. In each year except 2015, the AI/AN population was observed to have higher disease prevalence than the white population (Fig. 1B).

### Determinants of chronic respiratory disease

**Fig. 2:**
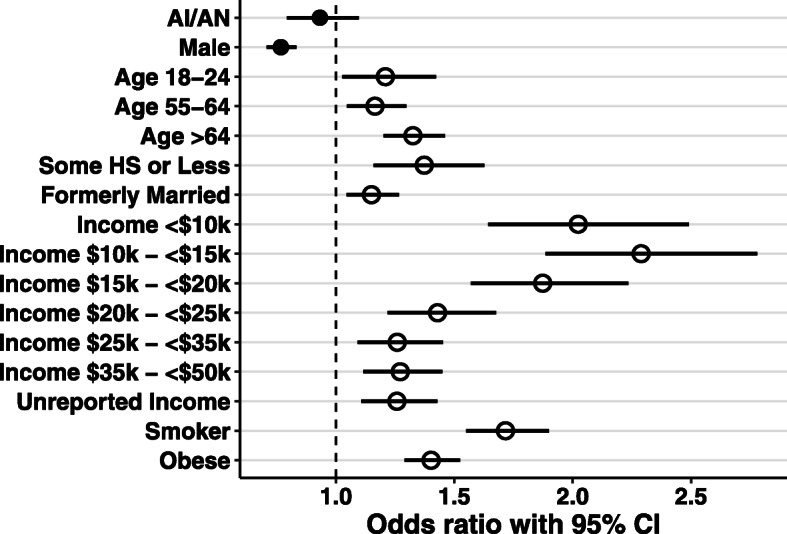
Determinants of chronic respiratory disease. Odds ratios with 95% CI are calculated from logistic regression performed on data from survey year 2017 for the 11 oversampled states. The AI/AN variable is not associated with chronic respiratory disease. Only significant covariates are shown. For covariates, closed circles represent variables negatively associated with disease. Open circles indicate variables positively associated with disease. Bars represent 95% CI. Reference levels for covariates are indicated in Table 1

To assess if being AI/AN contributed to having chronic respiratory disease, and to identify significant determinants of disease we performed logistic regression analysis. We present detailed results for 2017 when oversampling of the AI/AN population was undertaken but similar trends were found for other years. Among the 71,939 respondents included in the analysis of the 2017 data, 4501 (6.3%) respondents were AI/AN (Table 1). To select for covariates used in multivariable logistic regression, we included variables which were statistically different between healthy respondents and respondents with disease (Rao-Scott X^2^, *p* < 0.05). Being AI/AN was found to be not associated with chronic respiratory disease (OR, 0.93; 95% CI, 0.79–1.10) in multivariable logistic regression analysis after adjusting for covariates. By contrast, a number of behavioral and socioeconomic factors are positively associated with disease. Behavioral factors included being a smoker (OR, 1.72; 95% CI, 1.55–1.90) and being obese (OR, 1.40; 95% CI, 1.29–1.52). Socioeconomic determinants included having less than high school education (OR, 1.37; 95% CI, 1.16–1.63) and low income levels. Annual household income was generally inversely correlated with odds of having chronic respiratory disease ($35,000- < $50,000: OR, 1.27; 95% CI, 1.12–1.45; $25000- < $35,000: OR, 1.26; 95% CI, 1.09–1.45; $20000- < $25,000: OR, 1.43; 95% CI, 1.22–1.68; $15000- < $20,000: OR, 1.87; 95% CI, 1.57–2.24; $10000- < $15,000: OR, 2.29; 95% CI, 1.89–2.78; <$10,000: OR, 2.02; 95% CI, 1.64–2.49) (Fig. 2 and Additional File 1). Logistic regression was also performed on data from years 2011–2018 except 2015, when the adjusted prevalence of chronic respiratory disease was found to be not statistically different between AI/AN and white populations. We found race to be not associated with chronic respiratory disease in any survey year (Table 2) while significant covariates remained broadly the same (Additional File 1).

**Table 1:** Characteristics of analyzed control respondents and respondents with chronic respiratory disease (CRD) in 2017 BRFSS

	With CRD (%) ***n*** = 12,192	Control (%) ***n*** = 59,747	***p*** value ^a^
Race			
AI/AN	950 (1)	3551 (5)	<.01
White ^b^	11,242 (16)	56,196 (78)	
Sex			
Male	4972 (7)	28,614 (40)	<.001
Female ^b^	7220 (10)	31,133 (43)	
Age (Years)			
18–24	619 (1)	2879 (4)	<.001
25–34	1023 (1)	5669 (8)	
35–44 ^b^	1112 (2)	6504 (9)	
45–54	1536 (2)	8945 (12)	
55–64	2695 (4)	13,365 (19)	
≥ 65	5207 (7)	22,385 (31)	
Marital status			
Currently married ^b^	6437 (9)	36,708 (51)	<.001
Divorced, widowed, separated	3994 (6)	14,586 (20)	
Never married	1761 (2)	8453 (12)	
Education			
Less than high school	856 (1)	2456 (3)	<.001
High school graduate	3494 (5)	15,920 (22)	
Some college/technical	3958 (6)	18,318 (25)	
Four or more years of college ^b^	3884 (5)	23,053 (32)	
Income			
< $10 k	650 (1)	1591 (2)	<.001
$10 k - < $15 k	815 (1)	1919 (3)	
$15 k - < $20 k	997 (1)	2864 (4)	
$20 k - < $25 k	1151 (2)	4224 (6)	
$25 k - < $35 k	1253 (2)	5414 (8)	
$35 k - < $50 k	1639 (2)	8155 (11)	
$50 k - < $75 k	1605 (2)	9686 (13)	
≥ $75 k ^b^	2554 (4)	18,666 (26)	
Unreported income	1528 (2)	7228 (10)	
Access-to-care			
Inadequate ^b^	726 (1)	3107 (4)	<.01
Adequate	11,466 (16)	56,640 (79)	
Smoking status			
Smoker	2667 (4)	7937 (11)	<.001
Non-smoker ^b^	9525 (13)	51,810 (72)	
Obesity status			
Underweight	276 (0)	761 (1)	<.001
Normal ^b^	3283 (5)	18,450 (26)	
Overweight	3992 (6)	22,605 (31)	
Obese	4641 (6)	17,931 (25)	

^a^ Comparison of categorical variables between control respondents and respondents with CRD using Rao-Scott χ^2^ test

^b^ Indicates reference level used in multivariable logistic regression presented in Fig. 2

**Table 2:** Association between race and chronic respiratory disease for 2011–2018

	OR (95% CI)						
	Years^a^						
Race	2011	2012	2013	2014	2016	2017	2018
White	1 (Reference)	1 (Reference)	1 (Reference)	1 (Reference)	1 (Reference)	1 (Reference)	1 (Reference)
AI/AN	1 (0.83–1.19)	0.91 (0.77–1.08)	0.97 (0.80–1.18)	0.92 (0.79–1.07)	1.04 (0.88–1.22)	0.93 (0.79–1.1)	0.98 (0.82–1.17)

Adjusted for age, sex, income, marital status, education, access to care, smoking status, and body weight morbidity

^a^ 2015 was excluded from logistic regression analysis due to non-significant prevalence comparison between AI/AN and white populations

## Discussion

In this cross-sectional analysis using annual BRFSS surveys spanning 2011–2018, we observed higher prevalence of chronic respiratory disease in AI/AN respondents compared to non-Hispanic white respondents, and sought to characterize factors contributing to this disparity.

We found that being AI/AN was not independently associated with chronic respiratory disease. Adjusting for sociodemographic and behavioral covariates equalized the odds ratios for chronic respiratory disease between AI/AN and white populations. By contrast, socioeconomic factors such as low annual household income and educational attainment were strong determinants of chronic respiratory disease status. These findings were consistent for almost all years included in the analysis despite 2017 being the only year when the AI/AN population was oversampled. The exception was 2015 when the AI/AN sample could have been too small to reach statistical significance. Regardless, these consistent observations underscored the importance of low socioeconomic status in determining chronic respiratory disease status [27, 28], while further showing that the AI/AN characteristic retained no disparity after accounting for these covariates. These data also validate the effectiveness of such public health surveillance effort in capturing critical disease correlates.

Why might AI/AN people exhibit higher prevalence of chronic respiratory disease? It has been well documented that a greater proportion of AI/AN people live in poverty compared to other races. In the most recent 5-year estimate (2014–2018) from the American Community survey, the median household income for the AI/AN population was $41,879 compared to $60,293 for the nation as a whole [29]. In the same period, 25.8% of AI/AN people lived below poverty level compared to 14.1% of the general population [30]. AI/AN levels of education attainment similarly lag behind that of other races [31] and AI/AN high school graduation rate was found to be lowest among all races/ethnicities [32]. Smoking prevalence was also highest for the AI/AN group compared to other race/ethnicities [33, 34]. The increased proportion of AI/AN people with low socioeconomic status is worrying. Low socioeconomic status has been consistently shown to be associated with worse disease outcome for both COPD [28, 35, 36] and asthma [37, 38],

In summary, our findings confirm that the AI/AN population still exhibits higher prevalence of chronic respiratory disease compared to the non-Hispanic white population. Positive disease covariates include established socioeconomic variables while the AI/AN racial characteristic is not independently associated with disease. Our study therefore recommends that efforts to further promote cooperative mobilization of public health and social service infrastructures may make progress to address this disease disparity.

### Strengths and limitations

Our study leveraged the oversampled data of the AI/AN population in the 2017 BRFSS to gain insight into the disparity of chronic respiratory disease. Despite 2017 being the only year with oversampling, our finding was consistent for all 7 years of the BRFSS where the AI/AN sample was found to be sufficient. However, we acknowledge a number of limitations in this study. Due to the cross-sectional nature of the BRFSS survey, temporal relationships and causality relationships of determinants cannot be established, some of which may also be bidirectional. This study relied on self-reported data in regards to health-risk behavior (smoking), disease status, and race classification. These types of self-classification might include nonrandom misclassification, and can present a limitation to the study, such as when self-reported smoking underestimates true smoking behavior [39]. However, some studies suggest that self-reported rates of smoking are generally reliable for large national US surveys [40, 41], and the present study confirms the expected observation that smoking status is positively associated with chronic respiratory disease. For self-reported asthma and COPD status, previous research has demonstrated general concordance between self-reported status and clinical diagnosis using spirometry [42, 43]. Lastly, our analysis was limited to covariates available from the BRFSS survey, which may fail to capture all confounders, some of which could be ethnoculturally unique to the AI/AN population.

## Conclusions

This study found that in the most recent years of the BRFSS, the AI/AN population experienced chronic respiratory disease disparity compared to the non-Hispanic white population. However, the AI/AN racial characteristic was not independently associated with disease status. This suggests that disproportionate socioeconomic challenges continue to exist for the AI/AN population. Our study therefore recommends cooperative mobilization of public health and social service infrastructures to address disease disparity.

## Supplementary Information

**Additional file 1.** Determinants of chronic respiratory disease for 2011–2018. This table lists detailed covariates and odds ratios obtained from logistic regression analyses for years 2011 through 2018.

## Data Availability

Annual BRFSS data is publicly available at: https://www.cdc.gov/brfss/annual_data/annual_data.htm

## Abbreviations

AI/AN: American Indian/Alaska Native
BRFSS: Behavior Risk Factor Surveillance System
CI: Confidence Interval
COPD: Chronic Obstructive Pulmonary Disease
OR: Odds Ratio
